# Butyrate Ameliorates ISO-Induced Cardiac and Intestinal Injury in Rats via Modulation of Bitter Taste Receptors (Tas2rs) and GPR41/43 to Inhibit NLRP3 Activation

**DOI:** 10.3390/nu18101530

**Published:** 2026-05-12

**Authors:** Tianxing Yu, Anqi Cao, Feng Zhu, Zhongwen Xie, Shanshan Hu, Daxiang Li

**Affiliations:** 1 State Key Laboratory of Tea Plant Germplasm Innovation and Resource Utilization, School of Tea Science, Anhui Agricultural University, Hefei 230036, China; 13979095263@163.com (T.Y.); caoanqi2019@126.com (A.C.); zhufeng_ahau@163.com (F.Z.); zhongwenxie@ahau.edu.cn (Z.X.); hshan1008@163.com (S.H.); 2 International Joint Laboratory on Tea Chemistry and Health Effects of Ministry of Education, Anhui Agricultural University, Hefei 230036, China

**Keywords:** butyrate, myocardial injury, gut–heart axis, Tas2r, NLRP3 inflammasome

## Abstract

**Background:** The gut microbiota and its metabolite short-chain fatty acids (SCFAs) regulate host physiology, but whether butyrate, a key SCFA, protects against myocardial injury via the gut–heart axis remains unclear. **Objectives:** This study aimed to investigate the cardioprotective effect of butyrate in a rat model of isoproterenol (ISO)-induced myocardial injury and to explore its underlying gut–heart mechanism. **Methods:** In this experimental study, male Sprague-Dawley rats received intragastric butyrate pre-treatment followed by ISO injection to induce myocardial injury. Cardiac function, myocardial remodeling, gut–heart homeostasis, intestinal barrier integrity, and the expression of Tas2r, GPR41/43, and NLRP3 pyroptosis pathway components were assessed. **Results:** Butyrate pre-treatment significantly restored cardiac function (LVEF increased by 19.67 units; 95% CI, 11.17–28.16; *p* < 0.001) and ameliorated electrophysiological abnormalities (QTc shortened by 63.21 ms; 95% CI, 45.45–80.97; *p* < 0.0001). Mechanistically, butyrate suppressed aberrant myocardial Tas2r signaling (Tas2r137 reduced by 1.06 units; 95% CI, 0.37–1.74; *p* < 0.01), upregulated GPR41/43, inhibited NLRP3 inflammasome activation (NLRP3 reduced by 1.23 units; 95% CI, 0.13–2.33; *p* < 0.05), and repaired intestinal barrier integrity, thereby reducing bacterial translocation and secondary injury. **Conclusions:** Butyrate ameliorates ISO-induced myocardial injury through a simultaneous gut–heart mechanism, acting on both the cardiac Tas2r137/GPR41/43-NLRP3 pathway and intestinal barrier protection. These findings identify butyrate as a key functional molecule in gut–heart crosstalk and suggest its potential as a therapeutic agent for myocardial injury.

## 1. Introduction

The gut–heart axis is a bidirectional regulatory network formed by the gut microbiome and its metabolites, intestinal barrier function, and immune function, through multi-pathway signal communication with the cardiovascular system [1]. Dysregulation of gut microbiota homeostasis is a key risk factor for the initiation and progression of various cardiovascular diseases, whereas pathological changes in the cardiovascular system can, in turn, reciprocally regulate gut microbiota composition and barrier function, thereby establishing a vicious pathological cycle [2].

Butyrate exerts cardioprotective effects through several mechanisms. First, it inhibits histone deacetylase (HDAC) activity, promotes regulatory T cell (Treg) differentiation, suppresses the NF-κB inflammatory pathway, and reduces the release of pro-inflammatory cytokines such as TNF-α and IL-6 [3,4,5]. Second, butyrate serves as an energy substrate for cardiomyocytes, enhances mitochondrial respiratory chain complex activity, and improves energy homeostasis under ischemic or hypoxic conditions [6]. Third, it activates GPR41/43 receptors, which promotes endothelial nitric oxide release, induces vasodilation, and lowers peripheral resistance [7]. Finally, butyrate upregulates Bcl-2, downregulates Bax, inhibits the Caspase-3 apoptotic cascade, and thereby reduces cardiomyocyte death induced by catecholamine overload or ischemia/reperfusion injury [8]. Furthermore, receptor activation represents a central pathway in SCFA-mediated gut–heart crosstalk. SCFAs specifically activate G protein-coupled receptors, among which GPR41/FFAR3 and GPR43/FFAR2 are widely distributed in cardiomyocytes, vascular endothelial cells, and immune cells. Upon activation, they regulate the cAMP, MAPK, and NF-κB signaling pathways, mediating anti-inflammatory, anti-apoptotic, and vascular homeostasis effects [9,10]. In addition, butyrate specifically activates the GPR109A receptor to inhibit inflammatory cascades in macrophages and myocardial tissue [11].

Bitter taste receptors (TAS2Rs) are widely distributed in intestinal tissues. Acting not only as chemical sensors for luminal contents, they also play a critical role in maintaining intestinal homeostasis by regulating diverse processes including hormone secretion, intestinal motility, and immune defense [12]. As key components of the innate immune system, intestinal TAS2Rs directly participate in host–microbiota interactions [13]. Upon activation by bitter ligands, TAS2R10 and TAS2R43 expressed in Paneth cells promote the release of antimicrobial peptides such as α-defensins, β-defensins, and REG3α, thereby inhibiting the growth of pathogenic bacteria [14]. Activation of TAS2R43 in goblet cells upregulates the expression of mucin MUC2 and CLCA1, leading to thickening of the intestinal mucus layer [15]. Stimulation of TAS2Rs in tuft cells triggers an IL-25-mediated type 2 immune response, facilitating the expulsion of pathogens, including parasites [16]. Furthermore, TAS2Rs participate in the regulation of local intestinal inflammatory responses: activation of TAS2R16 suppresses LPS-induced NF-κB nuclear translocation and the expression of pro-inflammatory cytokines, exerting anti-inflammatory effects [17].

Butyrate, a gut microbial metabolite, can enter the circulation and mediate gut–heart crosstalk. However, in isoproterenol (ISO)-induced myocardial injury, it remains unclear whether butyrate exerts cardioprotection under sympathetic overactivation and whether Tas2rs participate in gut–heart communication. This study aims to address these questions.

## 2. Materials and Methods

### 2.1. Materials

Anti-T2R3/T2R137 (E-12), anti-TLR4, anti-Caspase-1, anti-ASC (N-15), and anti-TGFβ1 (3C11), anti-MCT1 and anti-TGF-β were from Santa Cruz Biotechnology (Dallas, TX, USA); anti-NLRP3 (D4D8T) was from Cell Signaling Technology (Danvers, MA, USA); anti-FOXP3, anti-Acetyl-Histone H3 and anti-H3 was from Jiangsu Qinke Biological Research Center Co., Ltd. (Affinity Biosciences, Changzhou, Jiangsu, China); anti-GPR41 and anti-Occludin were from Wuhan Sanying Biotechnology Co., Ltd. (Proteintech Group, Wuhan, Hubei, China); anti-GPR43 was from Thermo Fisher Scientific (Waltham, MA, USA); anti-MUC2 was from ABclonal Biotechnology Co., Ltd. (Wuhan, Hubei, China). Chemicals: Isoprenaline hydrochloride and tetramethyl ethylenediamine (TEMED) were purchased from Sigma (St. Louis, MO, USA). Sodium butyrate (C4H7NaO2; purity ≥98%) was purchased from Shanghai Macklin Biochemical Co., Ltd. (Macklin, Shanghai, China).

### 2.2. Animals

A total of 30 specific pathogen-free (SPF)-grade male Sprague-Dawley (SD) rats (6 weeks old, body weight 180–220 g) were purchased from Spefu (Beijing) Biotechnology Co., Ltd. (Beijing, China). The rats were randomly divided into three groups, and the formal experiment was performed after a one-week acclimatization period. Before the experiment, the animals were housed 4 = four per cage in a room with controlled temperature (24 ± 2 °C) and humidity (50 ± 5%), under a 12 h light/dark cycle, with free access to standard chow and water. All animal experimental procedures were approved by the Institutional Animal Care and Use Committee of Anhui Agricultural University (Ethics approval number: AHAU2022016). To minimize potential observer bias, group allocation was concealed from the investigator during drug administration. Echocardiographic and electrocardiographic recordings were analyzed by an independent operator blinded to group assignment. All tissue samples were coded, and personnel performing Western blot, qPCR, and histological analyses remained blinded until data analysis was completed. Full details are provided in the Author Checklist—Full.

### 2.3. ISO-Induced Myocardial Injury Model in Rats

Isoproterenol (ISO), a catecholamine, at high doses induces myocardial injury through auto-oxidation and lipid peroxidation [18]. After one week of acclimation, 30 rats were randomly divided into three groups (n = 10 each): control (CON, saline); ISO group (85 mg/kg ISO subcutaneously once daily for 2 days; ISO was freshly dissolved in sterile 0.9% normal saline immediately before administration, with a standardized subcutaneous injection volume of 1 mL/kg body weight) [19]; and the butyrate treatment group (BI), which received sodium butyrate (equivalent to 200 mg/kg butyric acid based on molecular weight conversion) by intragastric gavage. The group size of n = 10 was determined by a priori power analysis using G*Power software (version 3.1.9.7) with a two-tailed independent t-test (α = 0.05, power = 0.80). Since no published effect size for butyrate in an isoproterenol-induced myocardial injury model was available, we adopted a conservative large effect size of Cohen’s d = 1.5 based on the data from Hu et al. after standardization to a realistic magnitude. This calculation yielded a minimum of 10 animals per group. This sample size is larger than the n = 6 per group successfully used in Yu et al. and thus provides more conservative statistical power. This dose range is supported by previous studies demonstrating significant anti-inflammatory and cardioprotective effects of butyrate in rat myocardial injury models with good biosafety [20,21]. After 7 days of intragastric treatment, ISO-induced myocardial injury was performed. All rats except CON received ISO to induce myocardial injury; saline served as a vehicle. Twenty-four hours after the second ISO injection, rats were anesthetized with isoflurane (1.5%) for ECG and left ventricular ultrasound measurements. Thereafter, rats were deeply anesthetized with 3% isoflurane (depth of anesthesia confirmed by the absence of toe pinch reflex) and euthanized by rapid exsanguination via the abdominal aorta. Subsequently, myocardial tissues were collected in RNA preservation solution and liquid nitrogen, then stored at −80 °C for further analysis.

### 2.4. Rat ECG and Echocardiographic Measurements

Electrocardiogram (ECG) and echocardiographic measurements were performed according to previous studies [22,23]. On day 3, rats were anesthetized with isoflurane (1.5%, *v*/*v*) while maintaining normal respiration. Cardiac function was assessed using a small animal ultrasound system (VINNO 6VET). M-mode images were obtained at the papillary muscle level from the left ventricular long-axis view to measure left ventricular ejection fraction (EF), fractional shortening (FS), and other echocardiographic parameters. After echocardiography, ECG signals were recorded using a ZL-620U medical signal acquisition and processing system. Needle electrodes were placed subcutaneously on the limbs in standard lead II configuration. ECG signals were acquired and analyzed using the system’s software. The following parameters were evaluated based on average signals: RR interval, P wave duration, QRS complex duration, Q-T interval, and corrected Q-T interval (Q-Tc = Q-T + 1.75 × (heart rate − 60)).

### 2.5. Determination of Bacterial Load in Rat Myocardial Tissue

To evaluate the relative content of gut microbiota crossing the intestinal barrier and entering cardiac tissue, real-time quantitative PCR was performed on rat myocardial tissue samples using the primer pairs 27F-1492R [24] and 1369F-1492R [25]. The primer sequences were as follows: 27F, AGAGTTTGATCMTGGCTCAG; 1369F, CGGTGAATACGTTCYCGG; 1492R, GGWTACCTTGTTACGACTT.

### 2.6. Real-Time Quantitative PCR

The qPCR procedure was performed according to a previous study [26]. Total RNA was extracted from an appropriate amount of rat left ventricular tissue using Total RNA Extraction Reagent. The 260/230 nm and 260/280 nm ratios and RNA concentration were measured using a micro-volume spectrophotometer. RNA (1 μg) was reverse-transcribed into cDNA using HiScript III RT SuperMix for qPCR (+gDNA wiper). Quantitative PCR was performed using ChamQ Universal SYBR qPCR Master Mix according to the manufacturer’s instructions, with specific primers for amplification and quantification of target genes. The primer sequences are detailed in Table S1.

### 2.7. Western Blotting

Rat tissue samples (~30 mg) were lysed in RIPA buffer, homogenized, and centrifuged at 12,000 rpm for 15 min at 4 °C. Protein concentration was determined using a BCA assay. Equal amounts of protein were separated by 10–12% SDS-PAGE and transferred onto PVDF/NC membranes, according to a previous study [27]. Membranes were blocked with 5% non-fat milk and incubated with primary antibodies overnight at 4 °C, followed by HRP-conjugated secondary antibodies. Protein bands were visualized using enhanced chemiluminescence (ECL) and quantified with Image J software, normalized to β-actin or GAPDH.

### 2.8. Statistical Analysis

Experimental data were analyzed using GraphPad Prism 9 software (GraphPad Software, Inc., San Diego, CA, USA). Results are presented as mean ± SEM. Comparisons among multiple groups were performed using one-way ANOVA followed by Tukey’s post hoc test. The value of *p* < 0.05 was considered statistically significant.

## 3. Results

### 3.1. Butyrate (i.g.) Ameliorates ISO-Induced Myocardial Systolic Dysfunction in Rats

**Figure 1:**
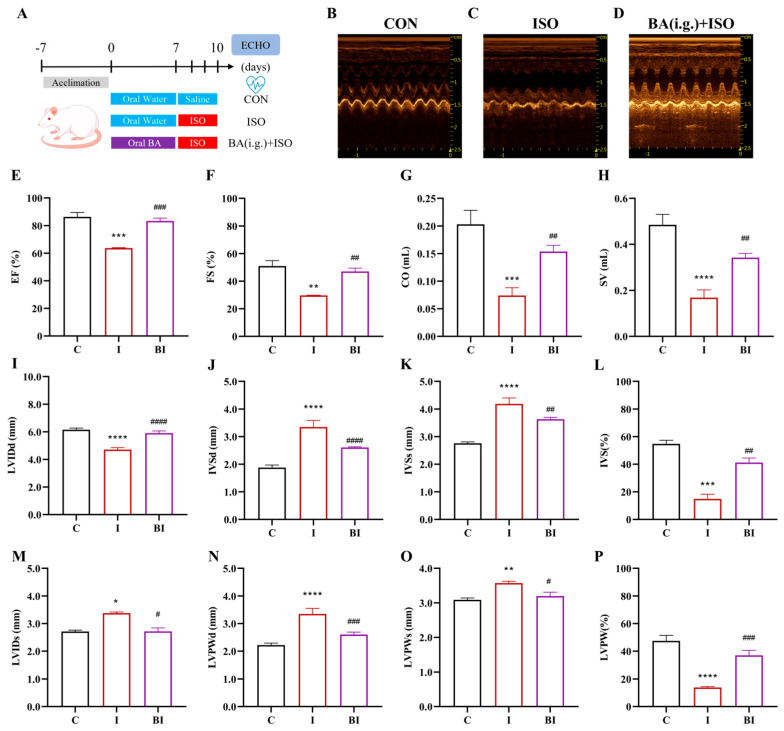
Butyrate ameliorates ISO-induced cardiac function impairment and structural abnormalities in rats. (**A**) Experimental timeline and grouping intervention protocol; (**B**–**D**) representative echocardiographic images of rats; (**E**–**H**) quantitative statistical results of EF, FS, CO, and SV, respectively; (**I**) LVIDd; (**J**) IVSd; (**K**) IVSs; (**L**) IVS%; (**M**) LVIDs; (**N**) LVPWd; (**O**) LVPWs; (**P**) LVPW%. All data are presented as mean ± SEM, n = 6. * *p* < 0.05, ** *p* < 0.01, *** *p* < 0.001, **** *p* < 0.0001 vs. CON group; ^#^ *p* < 0.05, ^##^ *p* < 0.01, ^###^ *p* < 0.001, ^####^ *p* < 0.0001 vs. ISO group. C, CON group; I, ISO group; BI, BA (i.g.) + ISO group.

To determine whether exogenous Butyrate gavage improves ISO-induced cardiac dysfunction, an intervention experiment was designed (Figure 1A). M-mode echocardiography showed that cardiac contractile amplitude was markedly reduced in the ISO group and significantly restored by Butyrate (Figure 1B–D). Quantitative analysis (Figure 1E–H) revealed that EF and FS were significantly decreased in the ISO group compared with the CON group, and both were significantly increased after Butyrate intervention; CO and SV were significantly decreased in the ISO group, and butyrate significantly reversed these changes. Collectively, butyrate significantly ameliorates ISO-induced systolic dysfunction.

Regarding cardiac structural parameters (Figure 1I–P): LVIDd was significantly decreased and LVIDs significantly increased in the ISO group, and butyrate significantly reversed these alterations. IVSd, IVSs, LVPWd, and LVPWs were all significantly increased (indicating myocardial hypertrophy) in the ISO group, and butyrate significantly suppressed these pathological thickenings. IVSd% and LVPWd% were significantly decreased in the ISO group, and butyrate significantly improved these indices. Thus, butyrate reverses ISO-induced left ventricular chamber deformation and pathological wall thickening and improves wall motion capacity.

### 3.2. Butyrate (i.g.) Ameliorates ISO-Induced ECG Abnormalities and Cardiac Hypertrophy in Rats

**Figure 2:**
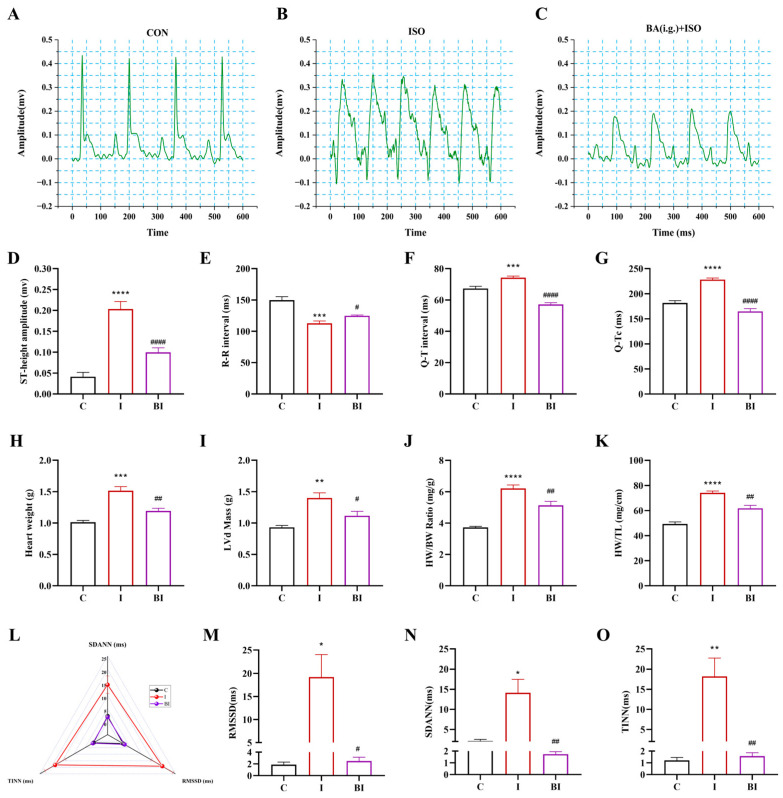
Butyrate ameliorates ISO-induced electrocardiographic abnormalities and heart weight alterations in rats. (**A**–**C**) Representative surface electrocardiogram waveforms of rats in the three groups; (**D**) ST segment elevation amplitude (mV); (**E**) R-R interval (ms); (**F**) Q-T interval (ms); (**G**) corrected Q-T interval (Q-Tc, ms); (**H**) heart weight (g); (**I**) left ventricular mass (LVMass, g); (**J**) heart weight/body weight ratio (HW/BW, mg/g); (**K**) heart weight/tail length ratio (HW/TL, mg/cm); (**L**) triangular radar chart of heart rate variability (HRV) indices; (**M**) SDANN (ms); (**N**) RMSSD (ms); (**O**) TINN (ms). All data are presented as mean ± SEM, n = 6. * *p* < 0.05, ** *p* < 0.01, *** *p* < 0.001, **** *p* < 0.0001 vs. CON group. ^#^ *p* < 0.05, ^##^ *p* < 0.01, ^####^ *p* < 0.0001 vs. ISO group. C, CON group; I, ISO group; BI, BA(i.g.)+ISO group.

To further assess the cardioprotective effects of butyrate, we evaluated electrocardiographic, autonomic, and morphological parameters (Figure 2). Compared with the CON group, the ISO group showed a significantly elevated ST segment, significantly prolonged R-R, Q-T, and Q-Tc intervals, and disordered ECG waveforms with ischemic changes; cardiac weight, left ventricular mass, HW/BW, and HW/TL were significantly increased. Butyrate intervention significantly reversed these abnormalities (ST segment, intervals, HW/BW, HW/TL), resulting in regular ECG rhythms and reduced cardiac hypertrophy (Figure 2D–K). HRV analysis revealed that SDANN, RMSSD, and TINN were significantly increased in the ISO group compared with the CON group, indicating autonomic dysfunction (radar plot). Butyrate significantly decreased these parameters, restoring autonomic balance (Figure 2M–O). These results provide direct evidence that butyrate protects cardiac function and improves myocardial remodeling at the electrophysiological, morphological, and autonomic levels.

### 3.3. Butyrate Ameliorates Histone Acetylation Imbalance, Oxidative Stress, Immune Dysregulation, and Tas2rs Disorder in the Myocardium of ISO-Induced Rats

**Figure 3:**
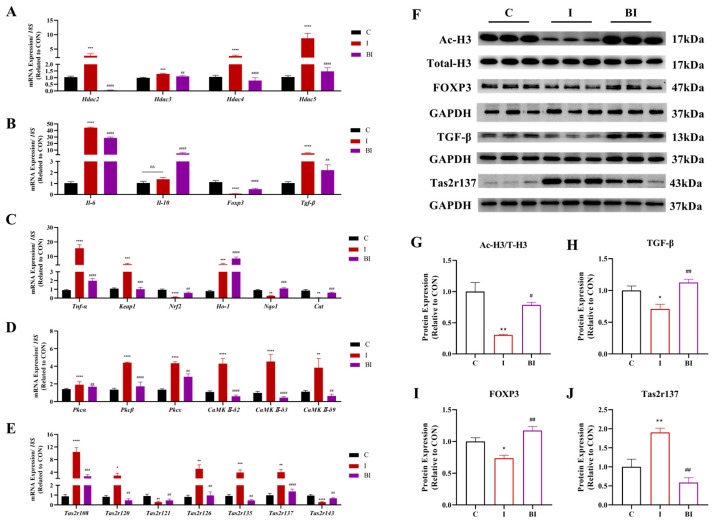
Effects of butyrate on histone modification, immune-related molecules and Tas2r signaling in ISO-induced rats. (**A**–**E**) mRNA expression levels of histone deacetylase genes (*Hdac2*, *Hdac3*, *Hdac4*, *Hdac5*), inflammation-associated genes (*Il-6*, *Il-10*, *Foxp3*, *Tgf-β*), oxidative stress-related genes (*Tnf-α*, *Keap1*, *Nrf2*, *Ho-1*, *Nqo1*, *Cat*), PKC/CaMK signaling genes (*Pkcα*, *Pkcβ*, *Pkcε*, *CaMKII-δ2*, *CaMKII-δ3* and *CaMKII-δ9*) and Tas2r family genes (*Tas2r108*, *Tas2r120*, *Tas2r121*, *Tas2r126*, *Tas2r135*, *Tas2r137*, *Tas2r143*); (**F**) representative Western blot images of Ac-H3, Total-H3, FOXP3, TGF-β and GAPDH; (**G**–**J**) quantitative analysis of Ac-H3/T-H3, TGF-β, FOXP3 and Tas2r137 protein expression. All data are presented as mean ± SEM (mRNA, n = 6; protein, n = 3). * *p* < 0.05, ** *p* < 0.01, *** *p* < 0.001, **** *p* < 0.0001 vs. CON group. ^#^ *p* < 0.05, ^##^ *p* < 0.01, ^###^ *p* < 0.001, ^####^ *p* < 0.0001 vs. ISO group. C, CON group; I, ISO group; BI, BA(i.g.)+ISO group.

Butyrate significantly inhibited ISO-induced gene expression of the myocardial deacetylase family and increased histone acetylation levels (Figure 3A,G). It also regulated the transcriptional expression of antioxidant and immune-related genes, significantly upregulating Nrf2 pathway genes and increasing TGF-β and FOXP3 protein expression in ISO-injured myocardium (Figure 3B,C,H,I). Further analysis revealed that butyrate markedly ameliorated the ISO-induced surge in myocardial Tas2rs family gene expression and suppressed Tas2r137 expression (Figure 3D,E,J).

### 3.4. Butyrate Ameliorates ISO-Induced Myocardial Inflammation via the Cardiac GPR41/43-NLRP3 Signaling Pathway

**Figure 4:**
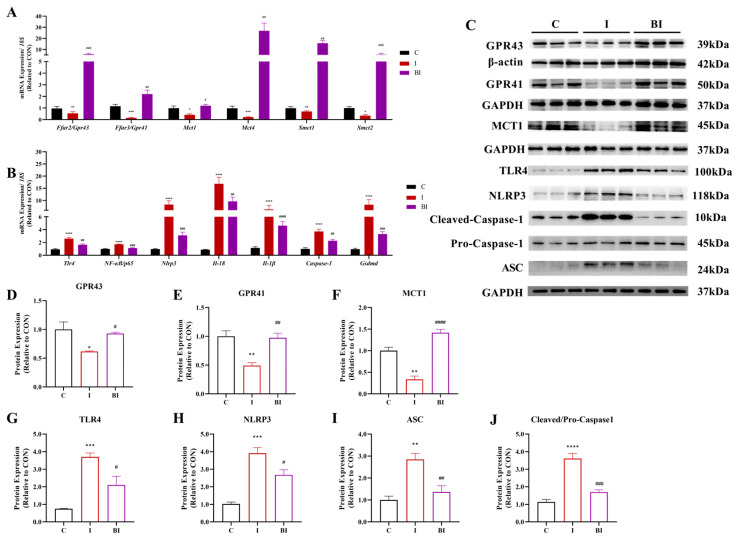
Effect of butyrate gavage on the expression of SCFA receptors/transporters and the NLRP3 inflammasome pathway in the myocardium of ISO-induced rats. (**A**) mRNA expression levels of SCFA receptor-related genes, including *Ffar2/Gpr43*, *Ffar3/Gpr41*, *Mct1*, and *Smct1/2*; (**B**) mRNA expression levels of intestinal inflammation-related genes, including *Tnf-α*, *NF-κB*, *Nlrp3*, *Il-1β*, *Il-18*, *Caspase-1*, and *Gsdmd*; (**C**) representative Western blot images of GPR43, GPR41, MCT1, TLR4, NLRP3, Cleaved-Caspase-1, Pro-Caspase-1, and ASC; (**D**–**J**) quantitative analysis of GPR43, GPR41, MCT1, TLR4, NLRP3, ASC, and Cleaved/Pro-Caspase-1 protein expression. All data are presented as mean ± SEM (mRNA, n = 6; protein, n = 3). * *p* < 0.05, ** *p* < 0.01, *** *p* < 0.001, **** *p* < 0.0001 vs. CON group. ^#^ *p* < 0.05, ^##^ *p* < 0.01, ^###^ *p* < 0.001, ^####^ *p* < 0.0001 vs. ISO group. C, CON group; I, ISO group; BI, BA (i.g.)+ISO group.

GPR41 and GPR43 are important environmental sensors in the gut, yet they are also present in myocardial tissue. ISO induced a surge of myocardial inflammation, accompanied by a significant decrease in *Gpr41*, *Gpr43*, *Mcts*, and *Smcts* family gene expression (Figure 4A). Butyrate gavage significantly reversed the ISO-induced reduction in SCFA receptor and transporter expression at both gene and protein levels (Figure 4D–F). Upon activation, GPR41 and GPR43 in myocardial tissue regulate downstream signaling; the NLRP3 inflammatory pathway was markedly activated in the myocardium of ISO-treated rats, and butyrate pretreatment significantly reduced myocardial NLRP3 inflammation levels (Figure 4G–J). These results indicate a cross-tissue protective effect of butyrate, which ameliorates myocardial inflammatory injury by regulating the GPR41/43-NLRP3 pathway in the heart.

### 3.5. Butyrate Ameliorates Histone Acetylation Imbalance, Oxidative Stress, Immune Dysregulation, and Tas2rs Disorder in the Intestine of ISO-Induced Rats

**Figure 5:**
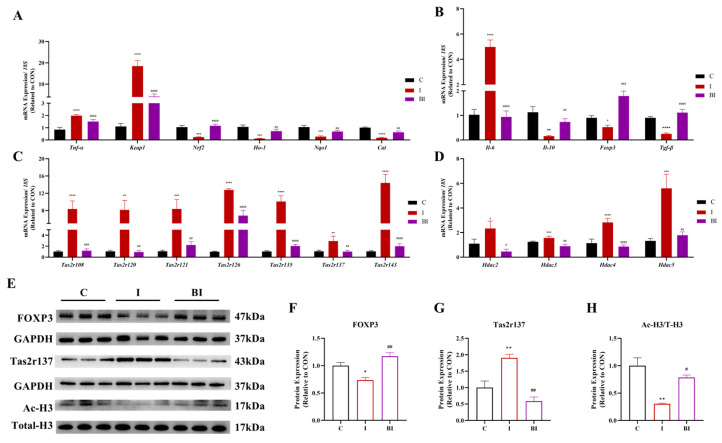
Effects of butyrate on intestinal oxidative stress, immune homeostasis, Tas2r signaling and histone acetylation in ISO-induced rats. (**A**) mRNA expression levels of intestinal oxidative stress- and inflammation-related genes, including *Tnf-α*, *Keap1*, *Nrf2*, *Ho-1*, *Nqo1*, and *Cat*; (**B**) mRNA expression levels of intestinal immune-related genes, including *Il-6*, *Il-10*, *Foxp3*, and *Tgf-β*; (**C**) mRNA expression levels of intestinal Tas2r family genes, including *Tas2r108*, *Tas2r120*, *Tas2r121*, *Tas2r126*, *Tas2r135*, *Tas2r137*, and *Tas2r143*; (**D**) mRNA expression levels of intestinal histone deacetylase genes, including *Hdac2*, *Hdac3*, *Hdac4*, and *Hdac5*; (**E**) representative Western blot images of FOXP3, Tas2r137, Ac-H3, Total-H3, and GAPDH; (**F**) Quantitative analysis of FOXP3 protein expression. (**G**) Quantitative analysis of Tas2r137 protein expression. (**H**) Quantitative analysis of Acetyl-Histone H3 (Ac-H3) relative to total Histone H3 (Total-H3) protein expression (Ac-H3/Total-H3 ratio). All data are presented as mean ± SEM (mRNA, n = 6; protein, n = 3). * *p* < 0.05, ** *p* < 0.01, *** *p* < 0.001, **** *p* < 0.0001 vs. CON group. ^#^ *p* < 0.05, ^##^ *p* < 0.01, ^###^ *p* < 0.001, ^####^ *p* < 0.0001 vs. ISO group. C, CON group; I, ISO group; BI, BA(i.g.)+ISO group.

The gut–heart axis plays a critical role in butyrate-mediated cardioprotection. Intestinal injury can further exacerbate myocardial damage. In ISO-induced myocardial injury rats, the imbalance of oxidative regulation and immune dysfunction in the intestine are detrimental to myocardial self-repair. Butyrate gavage significantly reduced the expression of inflammatory cytokines, increased the expression of Nrf2-mediated antioxidant genes and Foxp3-related immune-regulatory genes (Figure 5A,B), and elevated FOXP3 protein levels (Figure 5F). Various Tas2rs are distributed in the intestine, and bitter taste receptors are involved in intestinal homeostasis and other physiological processes. Our results showed that butyrate significantly restored the ISO-induced dysregulation of intestinal Tas2rs gene expression and markedly suppressed downstream NLRP3 pathway genes (Figure 5C,G). The transcription of these genes is regulated by histone acetylation; the ISO-induced activation of intestinal histone deacetylases was antagonized by the strong inhibitory effect of butyrate, thereby providing a basis for the transcription and translation of downstream signals (Figure 5D,H). These findings indicate that in the ISO-induced myocardial injury model, butyrate, as a key microbial metabolite, directly exerts intestinal stabilizing effects, which constitutes the intrinsic basis for its amelioration of myocardial injury.

### 3.6. Butyrate Ameliorates ISO-Induced Intestinal Injury and Barrier Dysfunction via the GPR41/43-NLRP3 Pathway

**Figure 6:**
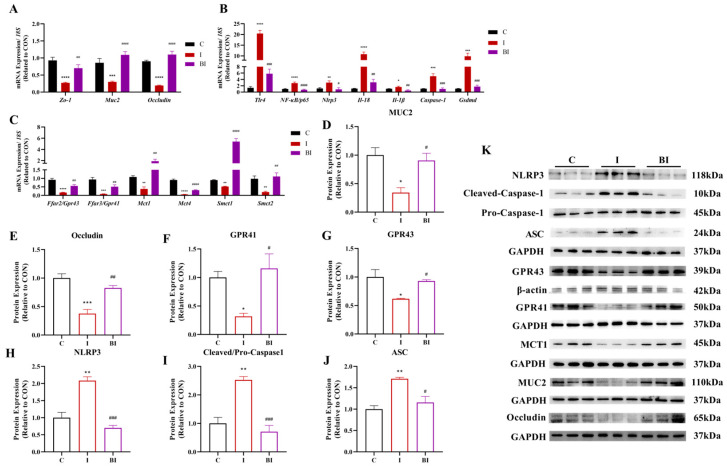
Effects of butyrate on intestinal barrier function, SCFA signaling and NLRP3 inflammasome in ISO-induced myocardial injury rats. (**A**) mRNA expression levels of intestinal barrier-related genes, including *Zo-1*, *Muc2*, and *Occludin*; (**B**) mRNA expression levels of intestinal inflammation and pyroptosis-associated genes, including *Tlr4*, *NF-κB*/*p65*, *Nlrp3*, *Il-18*, *Il-1β*, *Caspase-1*, and *Gsdmd*; (**C**) mRNA expression levels of intestinal SCFA receptor and transporter genes, including *Ffar2/Gpr43*, *Ffar3/Gpr41*, *Mct1*, *Mct4*, *Smct1*, and *Smct2*; (**D**–**J**) Quantitative analysis of intestinal protein expression levels of MUC2, Occludin, GPR41, GPR43, NLRP3, Cleaved/Pro-Caspase-1, and ASC, respectively; (**K**) representative Western blot images of all detected intestinal proteins. All data are presented as mean ± SEM (mRNA, n = 6; protein, n = 3). * *p* < 0.05, ** *p* < 0.01, *** *p* < 0.001, **** *p* < 0.0001 vs. CON group. ^#^ *p* < 0.05, ^##^ *p* < 0.01, ^###^ *p* < 0.001, ^####^ *p* < 0.0001 vs. ISO group. C, CON group; I, ISO group; BI, BA(i.g.)+ISO group.

To investigate the mechanism underlying the Tas2rs downstream signaling pathway and the repair of the intestinal barrier structure, we measured the expression of intestinal barrier and mucin genes and proteins. ISO caused significant intestinal barrier damage, whereas butyrate treatment improved the expression of tight junction proteins and mucins (Figure 6A,D,E). The stability of the intestinal barrier is reflected by a significant reduction in bacterial load in the myocardium, indicating that butyrate improves intestinal barrier function to prevent bacterial translocation caused by ISO injury, thereby counteracting the exacerbation of myocardial injury (Figure S1). Further analysis revealed that ISO induced impairment of SCFA sensing and transport in the intestine, as evidenced by the significantly reduced expression of GPR41/43 receptors at both gene and protein levels, and butyrate significantly restored their expression (Figure 6C,F,G). The downstream NLRP3 pathway proteins were also markedly inhibited by butyrate (Figure 6B,H,J), confirming that butyrate exerts its protective effect in gut–heart communication via the GPR41/43-NLRP3 signaling pathway.

## 4. Discussion

A dietary intake of vegetables, fruits and other plant-based foods is rich in indigestible carbohydrates such as resistant starch and dietary fiber, which cannot be directly absorbed and utilized by the host [28]. Upon reaching the colon, these nutrients are fermented and decomposed by the gut microbiota, producing key active metabolites including SCFAs [29]. In recent years, a large body of evidence has confirmed that gut microbiota dysbiosis is a critical risk factor for the development and progression of cardiovascular diseases, and the pathological process of myocardial injury is closely associated with impaired intestinal barrier function, disrupted gut microbiota composition, and abnormal alterations in microbial metabolites in the host [1].

This study shows that exogenous butyrate exerts dual gut–heart protection: it ameliorates ISO-induced myocardial dysfunction, electrophysiological disorders, hypertrophy, oxidative stress, and inflammation, and enhances colonic barrier stability, immune balance, and Tas2rs homeostasis. Mechanistically, butyrate upregulates GPR41/43, inhibits NLRP3, and increases histone acetylation via HDAC inhibition. It should be noted that while the upregulation of GPR41/43 likely involves both direct agonism and indirect transcriptional enhancement via HDAC inhibition, the restoration of Tas2r homeostasis is more plausibly interpreted as an indirect consequence of butyrate’s anti-inflammatory and epigenetic effects. Bitter taste receptor signaling is highly sensitive to the inflammatory milieu, and its dysregulation during myocardial injury can further amplify myocardial damage, potentially through the NLRP3 inflammasome pathway. By suppressing inflammation and oxidative stress, butyrate indirectly normalizes Tas2r expression, thereby interrupting the vicious cycle in which Tas2r signaling disturbance exacerbates myocardial injury. This logic supports the rationale for investigating the Tas2r–NLRP3 axis as a mechanistically relevant pathway through which butyrate exerts its gut–heart protective effects. Previous studies support that butyrate is negatively correlated with myocardial injury [30]. A recent comprehensive review further systematized these cardioprotective mechanisms, delineating that butyrate influences cardiovascular health through a “trinity” mode of action encompassing GPCR modulation (GPR41/43/109A), HDAC inhibition (classes I–IV), and peroxisome proliferator-activated receptor (PPAR) activation, thereby concurrently regulating inflammation, oxidative stress, and energy metabolism in conditions ranging from hypertension and atherosclerosis to heart failure [31]. These multimodal actions position butyrate as a uniquely versatile gut-derived metabolite with broad therapeutic potential across the cardiovascular disease spectrum.

At the myocardial electrophysiological level, butyrate significantly reversed ISO-induced prolongation of the Q-T interval, Q-Tc abnormalities, and ST-segment deviation, indicating its antiarrhythmic effect. This provides new evidence for the conclusion that SCFAs ameliorate myocardial electrophysiological disorders [32]. Notably, recent experimental evidence has extended this electrophysiological protection to the autonomic nervous system level: butyrate attenuates sympathetic activation in rats with chronic heart failure by inhibiting microglial inflammation in the paraventricular nucleus, an effect accompanied by reduced renal sympathetic nerve activity and decreased plasma and cerebrospinal fluid norepinephrine levels [33]. This central sympatholytic mechanism may partly account for the antiarrhythmic effects observed in the present study, as sympathetic overdrive is a well-established trigger for QT prolongation and ventricular arrhythmogenesis. Furthermore, age-related gut microbiota dysbiosis and consequent reduction in SCFA levels have been functionally linked to impaired autonomic nervous system regulation and increased atrial fibrillation susceptibility in rats, reinforcing the concept that gut-derived SCFAs are physiological regulators of cardiac autonomic tone and arrhythmogenic substrate [34]. These findings collectively suggest that butyrate-mediated electrophysiological protection operates at multiple hierarchical levels, from direct cardiomyocyte ion channel modulation to central sympathetic outflow control. Mechanistically, butyrate inhibits myocardial oxidative stress and immune imbalance, downregulates the expression of pro-inflammatory cytokines such as TNF-α, IL-6, and IL-1β, and upregulates IL-10 and FOXP3, which is consistent with previous anti-inflammatory studies, confirming that it exerts cardioprotective effects by suppressing inflammation and oxidative damage [35]. Butyrate ameliorates ISO-induced colonic barrier injury by upregulating Occludin, ZO-1, and MUC2, confirming it as the key effector of ECG-mediated gut protection. This barrier-protective action has been corroborated across diverse injury models. In a chronic Toxoplasma gondii infection model, dietary butyrate supplementation significantly increased the thickness of the inner mucus layer and upregulated the expression of Occludin, ZO-1, and Claudin, while concurrently alleviating infection-induced intestinal cell senescence [36]. Moreover, butyrate was shown to protect the intestinal barrier by upregulating Fut2 (fucosyltransferase 2) expression via the MEK4-JNK signaling pathway, leading to increased fucosylation of epithelial glycoproteins and enhanced tight junction protein expression of Occludin and ZO-1 [37]. These convergent findings from pathologically distinct models reinforce the notion that butyrate-mediated barrier stabilization is a robust, broadly conserved property rather than a model-specific phenomenon. It also inhibits oxidative stress, inflammation, and NLRP3 activation, reducing pro-inflammatory cytokines and preventing gut-derived inflammation from exacerbating myocardial injury. The translational significance of butyrate-mediated gut barrier protection is highlighted by a landmark study demonstrating that GPR41/43 double knockout mice exhibit heightened gut permeability upon angiotensin II challenge, enabling the translocation of bacterial lipopolysaccharide into the systemic circulation and thereby exacerbating hypertension and cardiorenal fibrosis; TLR4 antagonism restored the protective phenotype, directly implicating the GPR41/43 axis in maintaining gut barrier integrity and preventing endotoxin-driven cardiovascular damage [38]. This gut barrier-centric mechanism provides a compelling explanation for how butyrate simultaneously protects the intestine and the heart: by strengthening the epithelial barrier, butyrate limits the systemic dissemination of pro-inflammatory microbial products that would otherwise fuel myocardial inflammatory injury during ISO challenge.

Butyrate exerts dual gut–heart protection via the GPR41/43-NLRP3 axis. It upregulates myocardial and colonic GPR41/43 and inhibits NLRP3 inflammasome activation (reducing NLRP3, ASC, Caspase-1, and IL-1β), consistent with prior reports [39]. For GPR41/43, butyrate is a well-established direct ligand, and its observed upregulation likely reflects both direct agonism and indirect transcriptional enhancement via HDAC inhibition. Extending the mechanistic understanding beyond NLRP3 inhibition alone, recent work employing molecular docking combined with in vivo rat models demonstrated that butyrate directly interacts with HDAC1, producing an approximately 40% reduction in HDAC1 expression and a 38% inhibition of NLRP3 inflammasome activation, with concomitant suppression of oxidative stress markers and promotion of M2 macrophage polarization [40]. Additionally, butyrate inhibits HDACs to increase histone acetylation and exert anti-inflammatory effects [41]. This epigenetic mechanism has been further refined by recent findings: butyrate restores the acetyl histone H3 (Lys27)-dependent transcriptome, thereby rescuing macrophage functions even in a persisting pro-inflammatory microenvironment and effectively breaking the vicious cycle of unresolved inflammation [42]. In the specific context of myocardial injury, the sodium butyrate-mediated suppression of HDAC2 has been shown to protect against doxorubicin-induced cardiomyocyte apoptosis by activating the PI3K/AKT signaling pathway, establishing HDAC2 isoform-specific inhibition as a pharmacologically relevant mechanism for butyrate’s cardioprotective actions [43]. Despite these insights, several limitations should be acknowledged. First, the experiments were conducted in an isoproterenol-induced rat model, which, although capable of mimicking certain features of myocardial injury caused by sympathetic overactivation, still differs from the complex etiology and comorbidity background of human disease; validation in multiple animal models and clinical cohorts remains warranted. Second, the preventive administration regimen primarily demonstrates the prophylactic protective effect of butyrate, while its therapeutic benefit when administered after injury onset has yet to be clarified. Finally, the unpleasant odor of native butyrate limits its clinical translation, and future exploration of optimized formulations or delivery strategies, as well as identification of suitable target populations, is needed.

## 5. Conclusions

**Figure 7:**
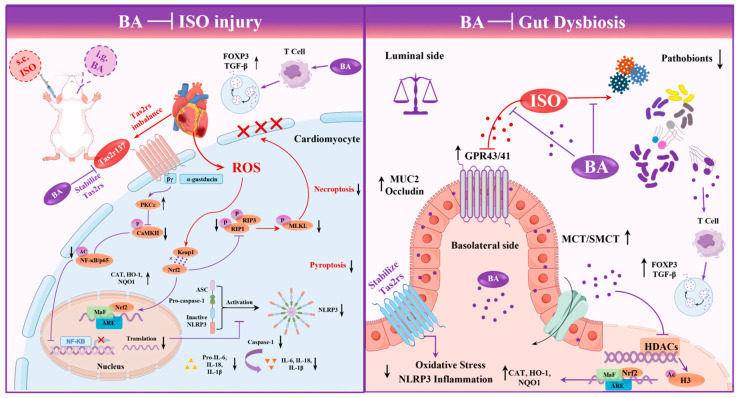
Graphical abstract. Illustration elements sourced from Figdraw (www.figdraw.com).

In summary, exogenous butyrate supplementation significantly ameliorates ISO-induced myocardial injury and intestinal barrier disruption in rats (Figure 7). Mechanistically, butyrate regulates Tas2r homeostasis in both the heart and intestine, upregulates GPR41/43, inhibits excessive NLRP3 inflammasome activation, and enhances histone acetylation via HDAC inhibition, thereby achieving coordinated gut–heart dual protection. These findings identify butyrate as a key gut-derived metabolite bridging intestinal homeostasis and myocardial resilience. As the ISO-induced model recapitulates features of human Takotsubo cardiomyopathy and early ischemic injury, understanding the preventive actions of butyrate may inform future therapeutic strategies for populations at high risk of sympathetic overactivation-induced cardiac injury.

## Data Availability

The original contributions presented in the study are included in the article; further inquiries can be directed to the corresponding author.

## Supplementary Materials

The following supporting information can be downloaded at: https://www.mdpi.com/article/10.3390/nu18101530/s1, Figure S1: Butyrate (i.g.) reduces bacterial burden in the myocardium of ISO-induced rats; Table S1: Primer sequences used for quantitative real-time PCR.

## Abbreviations

The following abbreviations are used in this manuscript:

BA	Butyrate
BCA	Bicinchoninic Acid
BI	Butyrate (intragastric) + Isoproterenol
C	Control
CON	Control
CO	Cardiac Output
ECG	Electrocardiogram
ECL	Enhanced Chemiluminescence
EF	Ejection Fraction
FS	Fractional Shortening
gDNA	genomic DNA
GAPDH	Glyceraldehyde-3-Phosphate Dehydrogenase
HDAC	Histone Deacetylase
HRV	Heart Rate Variability
HW/BW	Heart Weight/Body Weight
HW/TL	Heart Weight/Tail Length
IL	Interleukin
ISO	Isoproterenol
IVSd	Interventricular Septal Thickness in Diastole
IVSd%	Interventricular Septal Thickness in Diastole Percentage
IVSs	Interventricular Septal Thickness in Systole
LVIDd	Left Ventricular Internal Diameter in Diastole
LVIDs	Left Ventricular Internal Diameter in Systole
LVPWd	Left Ventricular Posterior Wall Thickness in Diastole
LVPWd%	Left Ventricular Posterior Wall Thickness in Diastole Percentage
LVPWs	Left Ventricular Posterior Wall Thickness in Systole
LVMass	Left Ventricular Mass
MAPK	Mitogen-Activated Protein Kinase
MCT	Monocarboxylate Transporter
MUC	Mucin
NC	Nitrocellulose
NF-κB	Nuclear Factor-kappa B
NLRP3	NOD-Like Receptor Family Pyrin Domain Containing 3
NO	Nitric Oxide
Nrf2	Nuclear Factor Erythroid 2-Related Factor 2
PCR	Polymerase Chain Reaction
qPCR	Quantitative Polymerase Chain Reaction
PVDF	Polyvinylidene Difluoride
Q-Tc	Corrected Q-T Interval
RR	R-R Interval
RIPA	Radio-Immunoprecipitation Assay
SCFAs	Short-Chain Fatty Acids
SD	Sprague-Dawley/Standard Deviation
SEM	Standard Error of the Mean
SDS-PAGE	Sodium Dodecyl Sulfate-Polyacrylamide Gel Electrophoresis
SPF	Specific Pathogen-Free
ST	ST Segment
SV	Stroke Volume
TEMED	Tetramethyl ethylenediamine
TGF-β	Transforming Growth Factor-Beta
TLR4	Toll-Like Receptor 4
TNF-α	Tumor Necrosis Factor-Alpha
Treg	Regulatory T Cell
ZO-1	Zonula Occludens-1

## References

[1] Rivera K., Gonzalez L., Bravo L., Manjarres L., Andia M.E. (2024). The Gut-Heart Axis: Molecular Perspectives and Implications for Myocardial Infarction. Int. J. Mol. Sci..

[2] Kondapalli N., Katari V., Dalal K.K., Paruchuri S., Thodeti C.K. (2025). Microbiota in Gut-Heart Axis: Metabolites and Mechanisms in Cardiovascular Disease. Compr. Physiol..

[3] Furusawa Y., Obata Y., Fukuda S., Endo T.A., Nakato G., Takahashi D., Nakanishi Y., Uetake C., Kato K., Kato T. (2013). Commensal microbe-derived butyrate induces the differentiation of colonic regulatory T cells. Nature.

[4] Dicks L.M.T. (2025). Butyrate Produced by Gut Microbiota Regulates Atherosclerosis: A Narrative Review of the Latest Findings. Int. J. Mol. Sci..

[5] Kespohl M., Vachharajani N., Luu M., Harb H., Pautz S., Wolff S., Sillner N., Walker A., Schmitt-Kopplin P., Boettger T. (2017). The Microbial Metabolite Butyrate Induces Expression of Th1-Associated Factors in CD4+ T Cells. Front. Immunol..

[6] Jiang X., Huang X., Tong Y., Gao H. (2020). Butyrate improves cardiac function and sympathetic neural remodeling following myocardial infarction in rats. Can. J. Physiol. Pharmacol..

[7] Pluznick J.L. (2016). Gut microbiota in renal physiology: Focus on short-chain fatty acids and their receptors. Kidney Int..

[8] Guo S., Zhang W., Cui X., Yin B. (2025). The bidirectional regulatory mechanism of gut microbiota metabolites on myocardial injury in heart failure from the perspective of the gut-heart axis: A review. Front. Microbiol..

[9] Pluznick J. (2014). A novel SCFA receptor, the microbiota, and blood pressure regulation. Gut Microbes.

[10] Liu M., Lu Y., Xue G., Han L., Jia H., Wang Z., Zhang J., Liu P., Yang C., Zhou Y. (2024). Role of short-chain fatty acids in host physiology. Anim. Models Exp. Med..

[11] Singh N., Gurav A., Sivaprakasam S., Brady E., Padia R., Shi H., Thangaraju M., Prasad P.D., Manicassamy S., Munn D.H. (2014). Activation of Gpr109a, receptor for niacin and the commensal metabolite butyrate, suppresses colonic inflammation and carcinogenesis. Immunity.

[12] Sternini C., Rozengurt E. (2025). Bitter taste receptors as sensors of gut luminal contents. Nat. Rev. Gastroenterol. Hepatol..

[13] Lee R.J., Cohen N.A. (2015). Taste receptors in innate immunity. Cell. Mol. Life Sci..

[14] Medapati M.R., Singh N., Bhagirath A.Y., Duan K., Triggs-Raine B., Batista E.L., Chelikani P. (2021). Bitter taste receptor T2R14 detects quorum sensing molecules from cariogenic Streptococcus mutans and mediates innate immune responses in gingival epithelial cells. FASEB J..

[15] Zheng X., Tizzano M., Redding K., He J., Peng X., Jiang P., Xu X., Zhou X., Margolskee R.F. (2019). Gingival solitary chemosensory cells are immune sentinels for periodontitis. Nat. Commun..

[16] Howitt M.R., Lavoie S., Michaud M., Blum A.M., Tran S.V., Weinstock J.V., Gallini C.A., Redding K., Margolskee R.F., Osborne L.C. (2016). Tuft cells, taste-chemosensory cells, orchestrate parasite type 2 immunity in the gut. Science.

[17] Zhou Z., Xi R., Liu J., Peng X., Zhao L., Zhou X., Li J., Zheng X., Xu X. (2021). TAS2R16 Activation Suppresses LPS-Induced Cytokine Expression in Human Gingival Fibroblasts. Front. Immunol..

[18] Boarescu P.-M., Boarescu I., BocȘAn I.C., Pop R.M., Gheban D., BulboacĂ A.E., Dogaru G., BolboacĂ S.D. (2019). Experimental model of acute myocardial infarction for evaluation of pr evention and rehabilitation strategies in cardiovascular diseases—A pilot study. BALNEO Res. J..

[19] Meeran M.F.N., Azimullah S., Adeghate E., Ojha S. (2021). Nootkatone attenuates myocardial oxidative damage, inflammation, and apoptosis in isoproterenol-induced myocardial infarction in rats. Phytomedicine.

[20] Hu X., Zhang K., Xu C., Chen Z., Jiang H. (2014). Anti-inflammatory effect of sodium butyrate preconditioning during myocardial ischemia/reperfusion. Exp. Ther. Med..

[21] Yu Z., Han J., Chen H., Wang Y., Zhou L., Wang M., Zhang R., Jin X., Zhang G., Wang C. (2021). Oral Supplementation with Butyrate Improves Myocardial Ischemia/Reperfusion Injury via a Gut-Brain Neural Circuit. Front. Cardiovasc. Med..

[22] Nekvindova E., Hrdlicka J., Boros A., Slegrova M., Kvasilova A., Skop V., Halberstat J., Holzerova K., Neckar J., Sedmera D. (2025). Electrical Remodeling of Pressure Overloaded Rat Heart Is Attenuated if Imposed During Proliferative Cardiac Growth. Acta Physiol..

[23] Lin Z.H., Yu Q.L., Yi B.H., Xu W.C., He H.L., Huang K.Y., Zheng C., Wu S.J., Lin J.F. (2025). Protective Effects of Low-Intensity Pulsed Ultrasound on Cardiac Electrophysiological Function in a Rat Model of Ischemic Cardiomyopathy. J. Am. Heart Assoc..

[24] Jeremy A.F., Reich C.I., Sharma S., Weisbaum J.S., Wilson B.A., Olsen G.J. (2008). Critical Evaluation of Two Primers Commonly Used for Amplification of Bacterial 16S rRNA Genes. Appl. Environ. Microbiol..

[25] Hu Z., Meng H., Shi J.H., Bu N.S., Fang C.M., Quan Z.X. (2014). Community Size and Composition of Ammonia Oxidizers and Denitrifiers in an Alluvial Intertidal Wetland Ecosystem. Front. Microbiol..

[26] Wu H., Ye M., Liu D., Yang J., Ding J.W., Zhang J., Wang X.A., Dong W.S., Fan Z.X., Yang J. (2019). UCP2 Protects the Heart from Myocardial Ischemia/Reperfusion Injury via Induction of Mitochondrial Autophagy. J. Cell. Biochem..

[27] Caudal A., Tang X., Chavez J.D., Keller A., Mohr J.P., Bakhtina A.A., Villet O., Chen H., Zhou B., Walker M.A. (2022). Mitochondrial Interactome Quantitation Reveals Structural Changes in Metabolic Machinery in the Failing Murine Heart. Nat. Cardiovasc. Res..

[28] Ma H.K., Shen L., Zhao L.M., Ji H.F. (2025). Regulation of Resistant Starch, Non-Starch Polysaccharides, Resistant Oligosaccharides and Lignin on the Gut Microbiota and Association with Their Health Benefits. Food Funct..

[29] Tilg H., Kaser A. (2011). Gut Microbiome, Obesity, and Metabolic Dysfunction. J. Clin. Investig..

[30] Chen H.-C., Liu Y.-W., Chang K.-C., Wu Y.-W., Chen Y.-M., Chao Y.-K., You M.-Y., Lundy D.J., Lin C.-J., Hsieh M.L. (2023). Gut Butyrate-Producers Confer Post-Infarction Cardiac Protection. Nat. Commun..

[31] Xu Q., Liu X., Wang Z., Li X., Jiang Q., Xu M. (2025). Recent Advancements and Comprehensive Analyses of Butyric Acid in Cardiovascular Diseases. Front. Cardiovasc. Med..

[32] Seefeldt J.M., Homilius C., Hansen J., Lassen T.R., Jespersen N.R., Jensen R.V., Boedtkjer E., Bøtker H.E., Nielsen R. (2024). Short-Chain Fatty Acid Butyrate Is an Inotropic Agent With Vasorelaxant and Cardioprotective Properties. J. Am. Heart Assoc..

[33] Liu C., Yu H., Xia H., Wang Z., Li B., Xue H., Jin S., Xiao L., Wu Y., Guo Q. (2024). Butyrate Attenuates Sympathetic Activation in Rats with Chronic Heart Failure by Inhibiting Microglial Inflammation in the Paraventricular Nucleus. Acta Biochim. Biophys. Sin..

[34] Liu L., Yi Y., Yan R., Hu R., Sun W., Zhou W., Zhou H., Si X., Ye Y., Li W. (2024). Impact of Age-Related Gut Microbiota Dysbiosis and Reduced Short-Chain Fatty Acids on the Autonomic Nervous System and Atrial Fibrillation in Rats. Front. Cardiovasc. Med..

[35] Li M., van Esch B., Henricks P.A.J., Folkerts G., Garssen J. (2018). The Anti-Inflammatory Effects of Short Chain Fatty Acids on Lipopolysaccharide- or Tumor Necrosis Factor α-Stimulated Endothelial Cells via Activation of GPR41/43 and Inhibition of HDACs. Front. Pharmacol..

[36] Liu S., Zheng Y., Cui B., Yang J., Yuan B., Cao Y., Zhao Z., Sun Z., Wang Q., Yang X. (2025). Gut Microbiota-Derived Butyrate Alleviates the Impairment of Mice Intestinal Integrity Caused by Toxoplasma Gondii Infection. Life Sci..

[37] Zhang D.-D., Huang Z.-X., Liu X.-C., Ding X.-P., Li L., He Y., Ai Q., Li L.-Q., Bao L. (2025). Butyrate Protects the Intestinal Barrier by Upregulating Fut2 Expression via MEK4-JNK Signaling Pathway Activation. Pediatr. Res..

[38] R. R.M., Zheng T., Dinakis E., Xie L., Barbaro-Wahl A., Jama H.A., Nakai M., Paterson M., Leung K.C., McArdle Z. (2025). Gut Microbiota Metabolites Sensed by Host GPR41/43 Protect Against Hypertension. Circ. Res..

[39] Zuo K., Fang C., Liu Z., Fu Y., Liu Y., Liu L., Wang Y., Yin X., Liu X., Li J. (2022). Commensal Microbe-Derived SCFA Alleviates Atrial Fibrillation via GPR43/NLRP3 Signaling. Int. J. Biol. Sci..

[40] Ganggang K., Cheng G., Gang L., Wenwu Z., Di Z., Yan L., Baoshu X., Yiqin W. (2025). From Molecular Docking to Rat Models: Butyrate From a High-Fiber Diet Inhibits HDAC1 and NLRP3 Inflammasome to Alleviate Oxidative Stress and Inflammation After Spinal Cord Injury. J. Neurochem..

[41] Lin M.Y., de Zoete M.R., van Putten J.P., Strijbis K. (2015). Redirection of Epithelial Immune Responses by Short-Chain Fatty Acids through Inhibition of Histone Deacetylases. Front. Immunol..

[42] Singh K., Koroma A.K., Pandey R.K., Wang Y., Cheng J., Haas P., Aghapour M., Krug L., Hainzl A., Wlaschek M. (2025). Restoring Histone Acetylation Accelerates Diabetic Wound Repair by Improving the Spatiotemporal Dynamics of Macrophages. Adv. Sci..

[43] Liu J., Fu W., Wang X., Liang Z., Meng F. (2025). The Role of HDAC2 Inhibition in Cardioprotection against Doxorubicin-Induced Myocardial Injury. Front. Cardiovasc. Med..

