# Supplementary material for: Butyrate Ameliorates ISO-Induced Cardiac and Intestinal Injury in Rats via Modulation of Bitter Taste Receptors (Tas2rs) and GPR41/43 to Inhibit NLRP3 Activation
Source: Nutrients. 2026 May 12;18(10):1530. doi: 10.3390/nu18101530 (PMC13210049; doi:10.3390/nu18101530)
Supplement: Supplementary file 1 [file nutrients-18-01530-s001.zip › Supplementary Figure and Table.pdf]

Supplementary Figure 1

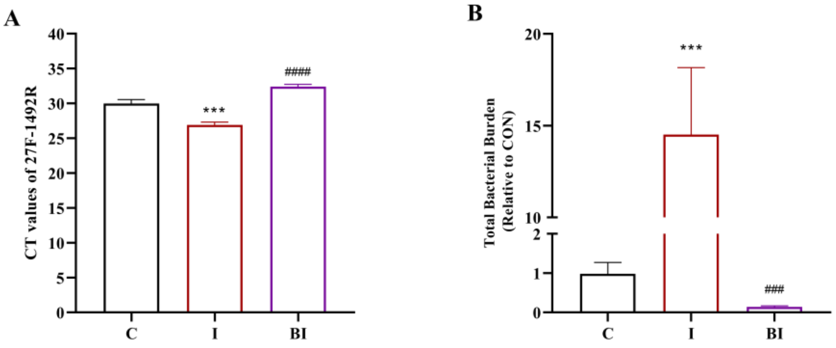

Fig. S1 Butyrate (i.g.) reduces bacterial burden in the myocardium of ISO-induced rats. (A) CT values of 27F-1492R; (B) Total bacterial burden in myocardial tissue (relative quantification using 1369F-1492R). All data are presented as mean ± SEM, n = 6. $^{***}P < 0.001$ vs. CON group. $^{\#\#\#}P < 0.001$, $^{\#\#\#\#}P < 0.0001$ vs. ISO group. C, CON group; I, ISO group; BI, BA (i.g.)+ISO group; BA, Butyrate.

Supplementary Table 1

Table S1 Primer sequences used for quantitative real-time PCR

Gene Name	Species	Orientation	Sequence (5'-3')
Tas2r108	Rat	Forward	GTTTGCCGCCTCAGTTGTTT
		Reverse	GGCCAAGCTGAACAGGATCT
Tas2r120	Rat	Forward	ACTGCTCTTGCCATCTCCAG
		Reverse	GGTCCCAAGCCAAGTGGTAA
Tas2r121	Rat	Forward	GGCTCTGGTCTCCTTCATCC
		Reverse	GGGCTTTTGTGCTAGGGTCT
Tas2r126	Rat	Forward	TACGCGCTGTCCTATGTGTC
		Reverse	GAAGGTGCCTCGGAACTTGA
Tas2r135	Rat	Forward	GTCCACAGGAGAAACGAGCA
		Reverse	ATCCATTGCCTACCACTGCC
Tas2r137	Rat	Forward	CTTACCACACCCACGACTCA
		Reverse	CACAGCATCCCAACAACCAC
Tas2r143	Rat	Forward	CACCCTGTCCTCTTTTGGCT
		Reverse	GGCAATGATGTTCCCGGTAG
Pkcα	Rat	Forward	AACCATCCGATCCACACTGAACC
		Reverse	AAGGAAAGGGAGCCCATGAAGTC
Pkcβ	Rat	Forward	GAGCAAGCAGAAGACCAAGACTATC
		Reverse	GTAACTTGAACCAGCCATCCACTC
Pkcε	Rat	Forward	TGGCTGTCTTTCACGATGCT
		Reverse	TTGTCTTTAGGGGCTTCGCC

CamkII-δ2	Rat	Forward	CCAGATGGGGTAAAGGAGTCAACTGAGAGCT
CamkII-δ3	Rat	Forward	AAAAGGAAGTCCAGTTCGAGTGTTCAGATGAT
CamkII-δ9	Rat	Forward	GTAAAGGAGCCCCAAACTACTGTAA
CamkII-δ2/3/9	Rat	Reverse	TCAGATGTTTTGCCACAAAGAGGTGCCTCCT
Trpm5	Rat	Forward	GGCCAATTGGAGAAGTTAACAG
		Reverse	AGGTGACACCAACAATGAACAG
Hdac2	Rat	Forward	ACTTGCCGTTGCTGATGCTT
		Reverse	TTGAACACCAGGCGCATGT
Hdac3	Rat	Forward	ACCGTGGCGTATTTCTACGAC
		Reverse	CCTGGTAAGGCTTGAAGACGA
Hdac4	Rat	Forward	CTCGCTGACCTCCGTGTTT
		Reverse	CTACACAGCCTACAGCCAGG
Hdac5	Rat	Forward	GATGCCGTTTTGAGTGCCTG
		Reverse	CCTGACATGCCATCCGACTC

Table S1 Primer sequences used for quantitative real-time PCR (continued)

Gene Name	Species	Orientation	Sequence (5'-3')
Tlr4	Rat	Forward	TGGCATTGTTCCTTTCCTGCC
		Reverse	TTCAGGGGGTTGAAGCTCAGAT
p65	Rat	Forward	GACGATCTGTTTCCCCTCAT
		Reverse	GCTTCTCTCCCCAGGAATAC
Nlrp3	Rat	Forward	CTGCATGCCGTATCTGGTTG
		Reverse	GCTGAGCAAGCTAAAGGCTTC
Il-1β	Rat	Forward	TTCAAATCTCACAGCAGCAT
		Reverse	CACGGGCAAGACATAGGTAG
Il-18	Rat	Forward	GCAGTAATACGGAGCATAAA
		Reverse	ATCCTTCACAGATAGGGTCA
Caspase-1	Rat	Forward	GGAGCTTCAGTCAGGTCCATC
		Reverse	GCGCCACCTTCTTTGTTCAG
Gsdmd	Rat	Forward	GGGGTGAAGATCGTGGATCA
		Reverse	AGGAGGCAGTAGGGCTTGAA
Il-6	Rat	Forward	GTTGCCTTCTTGGGACTGATG
		Reverse	ATACTGGTCTGTTGTGGGTGGT
Il-10	Rat	Forward	GCTCAGCACTGCTATGTTGC
		Reverse	TTGTCACCCCGGATGGAATG
Tgf-β	Rat	Forward	TACAACAGCACCCGCGACCG
		Reverse	TGCGTTGTTGCGGTCCACCA
Foxp3	Rat	Forward	TCACACGCATGTTCGCCTACTTC

		Reverse	CTCACTCCACTCGCACAAAGC
Zo-1	Rat	Forward	GCCCAGAGTGAAGGCAATTC
		Reverse	TCACAGTGTGGCAAGCGTAG
Muc2	Rat	Forward	GAAGCCAGATCCCGAAACCA
		Reverse	GCATTTGCGAGTTATCAG
Occludin	Rat	Forward	GAGGCTATGGCTATGGCTATGG
		Reverse	AGGAAGCGATGAAGCAGAAGG
18s	Rat	Forward	GGGTCGGGAGTGGGTAATTT
		Reverse	AGAAACGGCTACCACATCCAA
Ffar2	Rat	Forward	GAAGCCAAGCAAGGATCCAC
		Reverse	GGCCGTGAGGATCAAGGAAC
Ffar3	Rat	Forward	CGGCCCCTACAATATGTCCC
		Reverse	AGGAGCTGATGAAAGTCGGC

Table S1 Primer sequences used for quantitative real-time PCR (continued)

Gene Name	Species	Orientation	Sequence (5'-3')
Mct1	Rat	Forward	CTGGTCGGTCGTGTAGGTG
		Reverse	GCGGCGAAAACCTAGTATCGTT
Mct2	Rat	Forward	AATCTGGAGGCTGCTCTACC
		Reverse	ATGTTTCTCTTGGCTGTTGTCAG
Mct4	Rat	Forward	GCAGTAATACGGAGCATAAA
		Reverse	ATCCTTCACAGATAGGGTCA
Smct1	Rat	Forward	CTTCGCCAACTCTGTGGGAG
		Reverse	ACATCCAGTCTGTCCCGTTG
Smct2	Rat	Forward	TTGGGGCATCCTTCCTCATC
		Reverse	CTCCCGTGTAGACAATCGTCTG
